# Supplementary material for: Fabrication of Functional bioMOF-100 Prototype as Drug Delivery System for Breast Cancer Therapy
Source: Pharmaceutics. 2022 Nov 15;14(11):2458. doi: 10.3390/pharmaceutics14112458 (PMC9699488; doi:10.3390/pharmaceutics14112458)
Supplement: Supplementary file 1 [file pharmaceutics-14-02458-s001.zip › pharmaceutics-2016669-supplementary.pdf]

SUPPORTING INFORMATION

Fabrication of Functional bioMOF-100 Prototype as Drug Delivery System for Breast Cancer Therapy

Renata Carolina Alves [1,*], Richard Perosa Fernandes [2], Renan Lira de Farias [3], Patricia Bento da Silva [4], Raquel Santos Faria [4], Christian Rafael Quijia [1], Regina Célia Galvão Frem [5], Ricardo Bentes Azevedo [4] and Marlus Chorilli [1]

[1] Department of Drugs and Medicines, School of Pharmaceutical Sciences, São Paulo State University (UNESP), Rodovia Araraquara Jau, Km 01—s/n—Campos Ville, Araraquara 14800-903, Brazil
[2] Department of Chemistry, Federal University of Mato Grosso (UFMT), Cuiabá 78060-900, Brazil
[3] Departament of Chemical, Pontifícia Universidade Católica do Rio de Janeiro, Rio de Janeiro 22451-900, Brazil
[4] Department of Genetics and Morphology, Institute of Biological Sciences, University of Brasilia (UnB), Campus Universitario Darcy Ribeiro—Asa Norte, Brasilia 70910-900, Brazil
[5] Chemistry Institute, São Paulo State University (UNESP), Campus Araraquara, Araraquara 14800-060, Brazil
* Correspondence: renata88_alves@hotmail.com

Figure S1. Analytical curve of the CCM in methanol solution obtained by the chromatographic method**.**

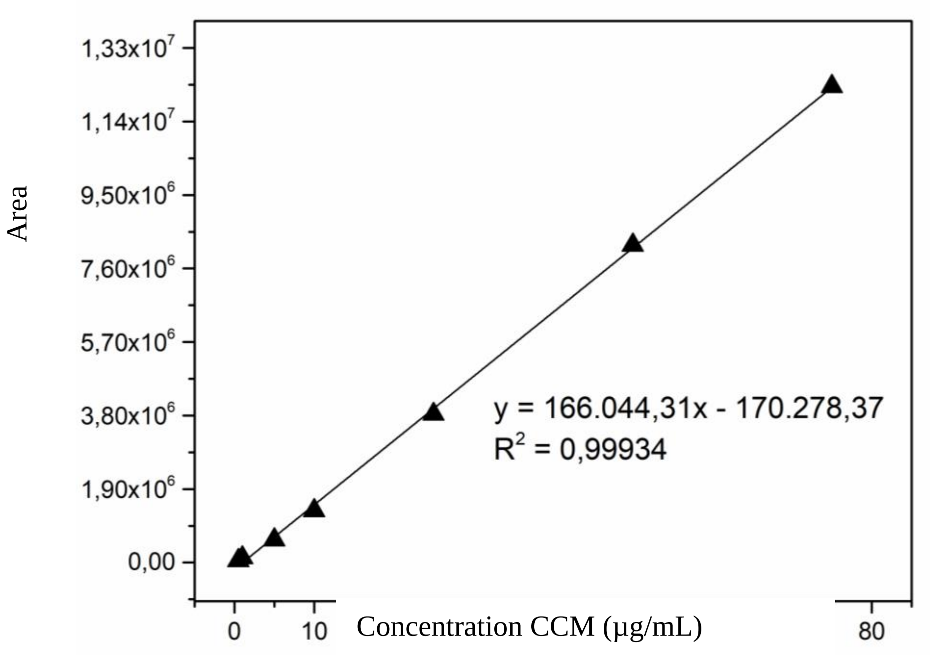

Figure S2. Optical polarized light microscopy of the solvated compounds: A) bioMOF-100, B) RCA.

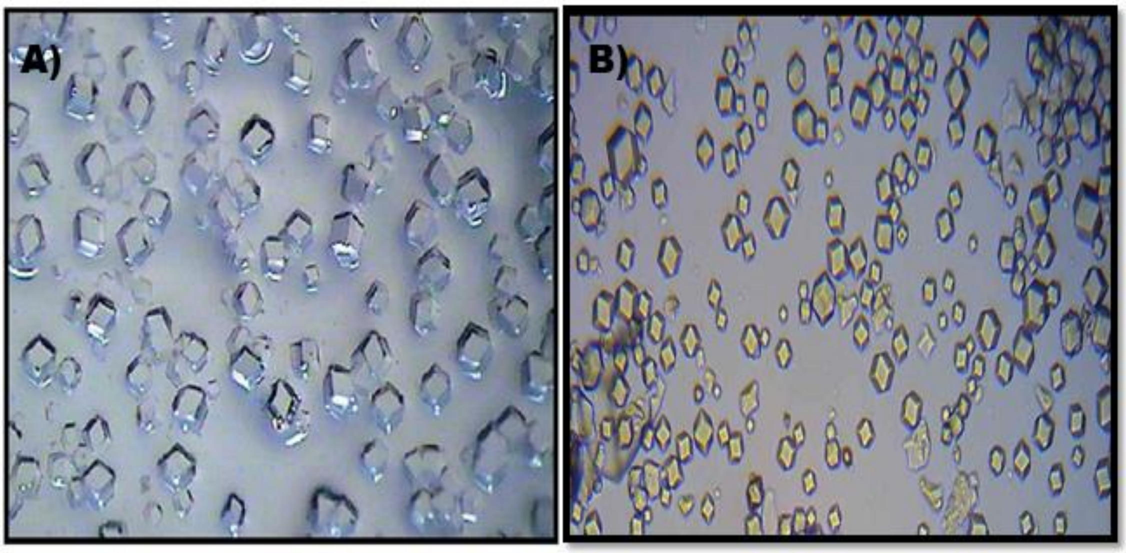

Figure S3. TGA-DSC curves of the RCA compound.

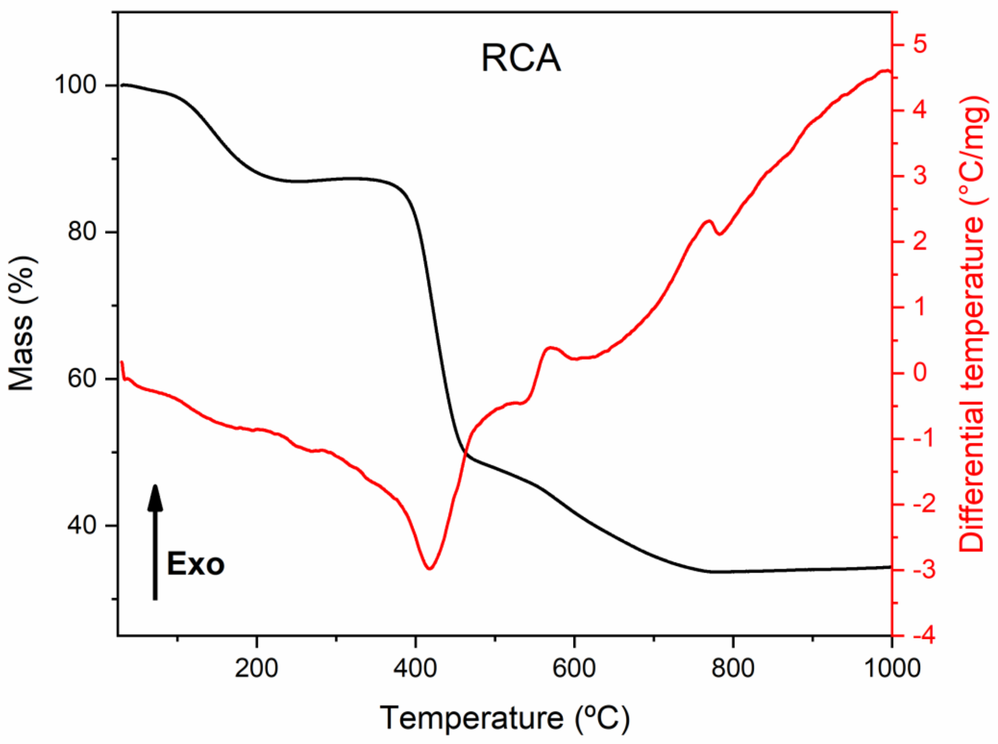

The TG curve shows mass losses in three stages, presenting two well-defined levels. The mass loss observed at the beginning of heating, between 30-113 °C (3.84%) can be attributed to the dehydration of the material. The second weight loss of 11.43%, observed between 113-374 °C, suggests the elimination of the DMF solvent and dimethylammonium cations, resulting from the thermal decomposition of DMF under solvothermic conditions. The third mass loss of 50.75%, observed between 374 and 782 °C, corresponds to the endothermic peak in the DSC curve at 417 °C, followed by two exothermic events at 571 and 767 °C, which may refer to the decomposition of the two ligands, followed by pyrolysis of the material.

Figure S4. Simulated crystalline structure of BioMOF-100, which has 85.5% voids/cell. The probe used consisted of a spherical molecule of $H_{2(g)}$, containing 1.2 Å radius.

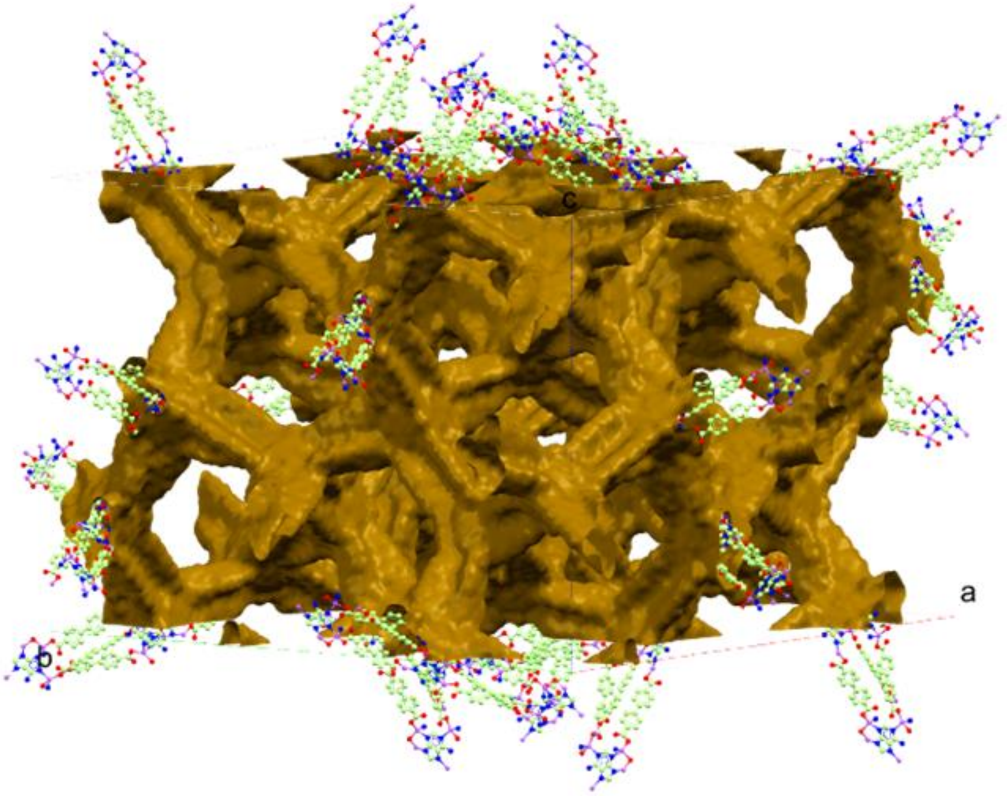

Figure S5. (a-b) accessible cavities referring to the existing solvent channels along the crystallographic direction [110].

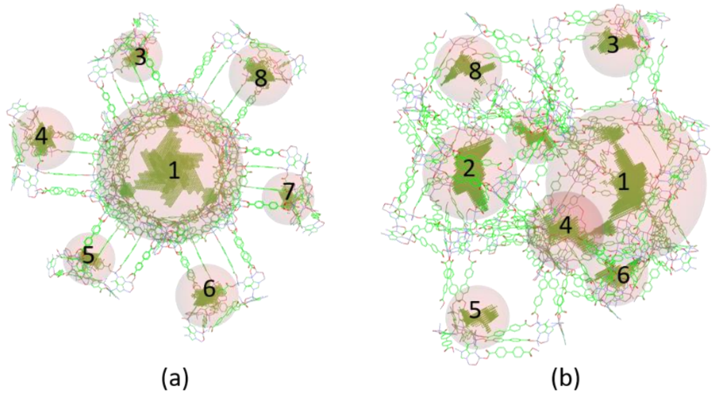

Figure S6. TG-DSC curves of the CCM@RCA-1D compound

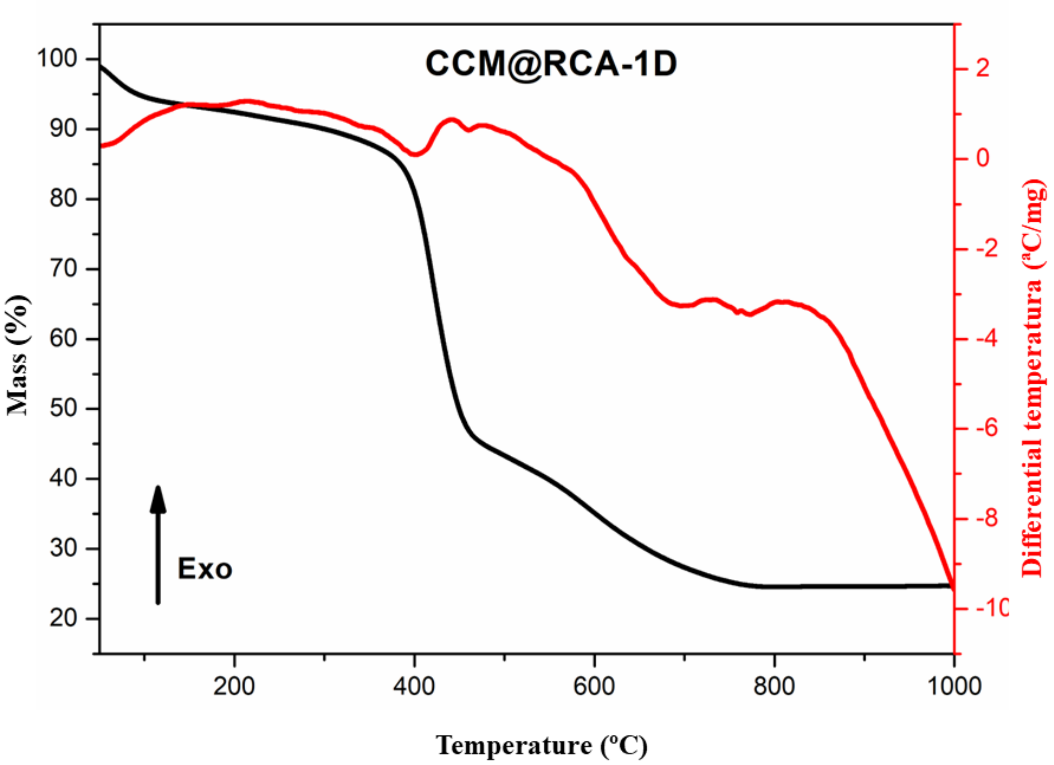

TG-DSC curves of the compound obtained after the CCM encapsulation test with a contact time of 1 day (CCM@RCA-1D). The TG curve presents mass losses in three stages, the first being 5.77% between 30-110 ºC, which can be attributed to the dehydration of the material. The second loss of 6.07% of mass, observed between 110-355 ºC, suggests the exit of solvent molecules and CCM molecules retained in the pores and/or on the surface of the material

Figure S7. Analytical curves of CCM in receptor medium obtained by the chromatographic method: A) at pH = 5.0 and B) at pH = 7.4.

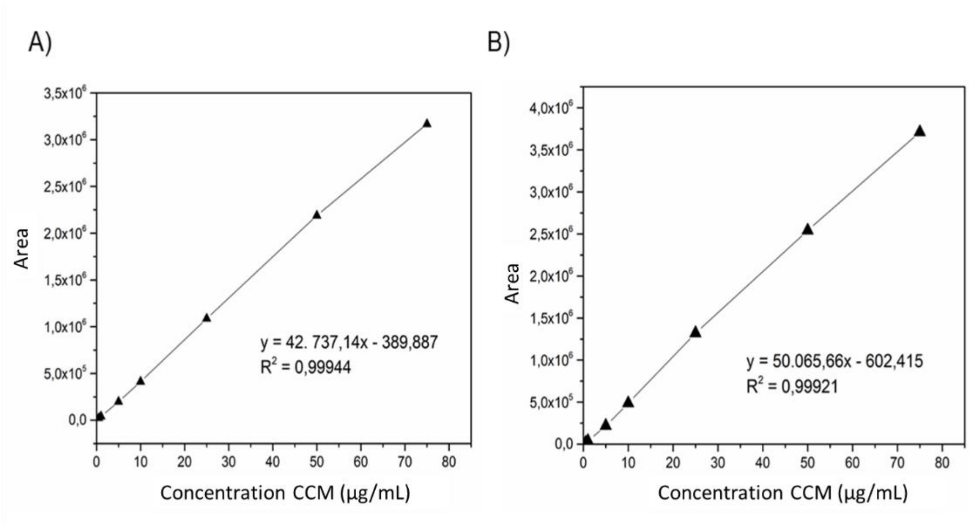

Table S1. Probability level relationship, conformer identification and RMSD.

Probability	Conformer	RMSD /Å
1	40	0,8902
1	14	0,9107
1	41	0,9509
1	12	0,9808
1	19	0,9974

Table S2. Percentage of CCM released from samples after 132 hours of experiment**.**

pH	Compounds	% CCM released
5,0	CCM@RCA-1D	28,14
	CCM@RCA-1D/FA	9,89
7,4	CCM@RCA-1D	9,58

CCM@RCA-1D/FA	6,55

Table S3. IC_{50} values of RCA precursors and series of compounds along with their IS values. IS*=$IC_{50\ (NIH/3T3)}$ / $IC_{50(MCF\text{-}7)}$; IS**= $IC_{50\ (NIH/3T3)}$ / $IC_{50(MDA\text{-}MB\text{-}321)}$ e IS***= $IC_{50\ (NIH/3T3)}$ / $IC_{50(4T1)}$.

**IC_{50} (µg/mL)**							
**Composts**	**NIH/3T3**	**MCF-7**	**IS***	**MDA-MB-231**	**IS****	**4T1**	**IS*****
Adenine	_	_	_	_	_	_	_
BPDC	_	_	_	_	_	_	_
CCM	25,87 ± 7,3	27,40 ± 6,23	0,94	26,63 ± 5,8	0,97	_	_
Acid Folic	_	_	_	_	_	_	_
RCA	42,85 ± 4,05	56,55 ± 7,01	0,76	84,51 ± 6,02	0,51	_	_
CCM@RCA-1D	34,19 ± 6,14	49,25 ± 6,05	0,70	69,00 ± 5,15	0,50	93,33 ± 9,9	0,37
CCM@RCA-1D/FA	_	35,61 ± 3,07	_	713,9 ± 9,38	_	97,64 ± 10,96	_
